# Recent advances in the understanding of autophagosome biogenesis

**DOI:** 10.12688/f1000research.22111.1

**Published:** 2020-03-26

**Authors:** Martin Graef

**Affiliations:** 1 Max Planck Institute for Biology of Ageing, Cologne, 50931, Germany; 2 Cologne Excellence Cluster on Cellular Stress Responses in Aging-Associated Diseases (CECAD), University of Cologne, Cologne, 50931, Germany

**Keywords:** Autophagy, autophagosome biogenesis, membrane contact sites, phospholipids

## Abstract

Autophagy is a conserved catabolic process critical for cell homeostasis with broad implications for aging and age-associated diseases. A defining feature of autophagy is the *de novo* formation of a specialized transient organelle, the double-membrane autophagosome. Autophagosomes originate from small vesicular precursors after rapid membrane expansion resulting in the engulfment of a broad spectrum of cytoplasmic cargoes within a few minutes for vacuolar or lysosomal degradation. Recent advances have provided exciting new insights into the molecular mechanisms underlying the assembly of autophagic membranes during autophagosome biogenesis. Specifically, the phospholipid biosynthesis activity of the endoplasmic reticulum and a dedicated membrane-tethering complex between nascent autophagosomes and the endoplasmic reticulum have emerged as key factors in autophagosome formation.

Macroautophagy, hereafter referred to as autophagy, is an intracellular mechanism critical for the maintenance of metabolic homeostasis and the removal of excess or dysfunctional cellular components in basal and stress conditions ^1^. Consistent with its central role, it has become clear that defects in autophagy are linked to aging and a broad spectrum of common age-associated diseases, including neurodegeneration, diabetes, and cancer ^2– 4^. Autophagy possesses an unparalleled scope of substrates ranging from protein aggregates to whole organelles and intracellular pathogens ^5^. The exceptional degradative capacity of autophagy is based on the formation of a specialized transient organelle, termed autophagosome, that surrounds and isolates substrates from the rest of the cytoplasm within a characteristic double-membrane structure. The biogenesis of an autophagosome begins with the nucleation of a small single-membrane vesicular structure, termed phagophore (or isolation membrane). The phagophore undergoes a stage of rapid membrane expansion and, within minutes, grows around the cargo in the shape of a large membranous cup. The cargo is topologically separated from the rest of the cytoplasm when the phagophore closes and divides its originally continuous single membrane into an inner and outer vesicle, the characteristic double-membrane structure of the autophagosome. The outer membrane of the autophagosome fuses with the lytic compartment (the vacuole in yeast and plants or the lysosome in mammals) to expose the inner vesicle and the engulfed cargo to degradation by resident hydrolases. The generated metabolites are recycled back to the cytoplasm. Thus, the life cycle of an autophagosome can be divided into five different stages: nucleation, expansion, closure of the phagophore, and maturation and fusion of the autophagosome with the lytic compartment ( Figure 1A) ^6– 8^. The origin of the autophagic membranes has been an outstanding question in the autophagy research field for many years.

**Figure 1.**
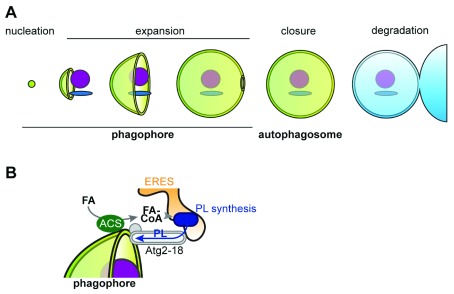
Autophagosome biogenesis. ( **A**) The stages of the life cycle of an autophagosome. ( **B**) Model for the role of local fatty acid (FA) activation, *de novo* phospholipid (PL) synthesis, and Atg2-mediated PL transfer during phagophore expansion. ACS, acyl-coenzyme A synthetase; CoA, coenzyme A; ERES, endoplasmic reticulum exit site.

A core set of autophagy proteins is essential for the biogenesis of autophagosomes and cooperates with a variety of cellular factors. Upon induction of autophagy, core autophagy proteins assemble in a hierarchical manner to initiate autophagosome formation. The biochemical events underlying the initial assembly have been characterized in considerable detail ^1, 9, 10^. In short, the Atg1/ULK kinase complex (composed of Atg1, Atg13, Atg17, Atg29, and Atg31 in yeast and ULK1/2, ATG13, ATG101, and FIP200 in mammalian cells) organizes into phase-separating supramolecular structures, resulting in auto-transactivation of the serine/threonine kinase activity of Atg1/ULK ^11, 12^. The Atg1/ULK kinase targets a number of downstream factors, including the essential autophagy protein Atg9 ^13^. Atg9 is a transmembrane protein that resides in small vesicles (Atg9 vesicles) that are derived from the Golgi apparatus and recycling endosomes ^14– 16^. Upon autophagy initiation, Atg9 vesicles are bound by the Atg1/ULK kinase complex and critically contribute to the nucleation of the phagophore ^17– 20^. In addition, the phosphatidylinositol 3 kinase complex I (PI3KI) (composed of Vps34, Vps15, Atg6, and Atg14 in yeast and VPS34, VPS15, Beclin1, and ATG14 in mammals) is recruited to the site of autophagosome formation and generates PI3P essential to autophagy ^9, 21^. PI3P is bound by the PROPPIN Atg18/WIPI proteins, which recruit Atg2/ATG2 to the phagophore membrane ^22– 24^. In addition, the action of the two Atg8 and Atg12 ubiquitin-like conjugation systems results in the covalent linkage of Atg8 proteins (Atg8 in yeast and LC3s and GABARAPs in mammals) to phosphatidylethanolamine within autophagic membranes with critical functions for phagophore elongation and substrate interactions ^25^.

The broad scope of substrates of autophagy suggests that cells form autophagosomes at many different sites in their cytoplasm. In yeast and mammals, autophagy protein assembly and formation of autophagosomes occur in close association with the intricate and dynamic network of the endoplasmic reticulum (ER), a seemingly universally conserved feature of autophagy ^26– 30^. Although it has been unclear what determines the specific site of autophagosome formation at the ER, autophagosomes do form at specialized subregions of the ER often in proximity to ER contact sites with other organelles, including mitochondria or plasma membrane in yeast and mammalian cells ^31– 33^. Specifically, in yeast, nascent autophagosomes are quantitatively linked to ER exit sites (ERESs) dedicated to the formation of COPII transport vesicles ^27, 28^. The function of ERESs is required for the assembly of the autophagy protein machinery downstream of the Atg1 kinase complex and, as a consequence, for the nucleation of the phagophore ^27, 34^. Strikingly, autophagic structures are stably tethered to ERESs throughout autophagosome biogenesis with one or two ERESs localizing to the rim of the expanding phagophore ^27, 28^. In line with this spatial arrangement, recent work has demonstrated that COPII vesicles are incorporated into autophagosomal membranes in yeast ^35^. In mammals, autophagosome formation initiates in proximity to PI3P-enriched membrane compartments connected to the ER, termed omegasomes, which closely enwrap nascent autophagosomes ^26^. Similar to yeast, ERESs and the ER-Golgi intermediate compartment (ERGIC) can be found in proximity to forming autophagosomes in mammalian cells, and COPII vesicles play an important role in the early stages of autophagosome biogenesis ^27, 36– 38^.

Close physical association of membrane-bound organelles is generally established by protein tethers, which organize these sites into membrane contact sites ^39^. Strikingly, the Atg2–Atg18 complex (ATG2A/B and WIPI4) has emerged as a tether for ER–phagophore contacts ^40– 43^. Cryo-electron microscopy (Cryo-EM) analyses have uncovered an extended rod-shaped conformation for Atg2 bridging the distance between two apposing membranes at membrane contact sites ^40, 43^. Indeed, *in vitro* experiments revealed the ability of the Atg2–Atg18 complex to tether membrane vesicles ^40, 42^. A C-terminal amphipathic helix is required for Atg2 localization to the phagophore while the N-terminus plays an important role for ER binding critical to the expansion of the phagophore in yeast ^42^. At the same time, the Atg2–Atg18 complex physically interacts with Atg9, confining it to the rim of the expanding phagophore and colocalizing it with ERESs ^27, 28, 41^. This spatial arrangement has been highly suggestive of phospholipid transfer reactions occurring at these ER–phagophore contacts. However, the molecular nature of these potential phospholipid transfer reactions had remained unclear. Excitingly, three independent studies have demonstrated that Atg2 itself displays phospholipid transfer activity *in vitro*: Atg2 can extract phospholipids from one vesicular membrane, bind them, and transfer them to a separate vesicular membrane ^44– 46^. This behavior is reminiscent of other membrane-tethering complexes. Indeed, Atg2 shares sequence and structural homology with the conserved membrane-tethering and lipid transfer protein Vps13 ^47^. Cryo-EM studies have revealed that Atg2 contains a cavity or groove along its extended conformation which can bind phospholipids indiscriminately in terms of headgroup or fatty acid chain composition ^44, 45^. The identification and first functional insights into the Atg2–Atg18 complex as a membrane tether and phospholipid transfer protein have provided a novel model of how at least parts of the membrane material required for the rapid expansion of the phagophore are transferred from the ER to the phagophore. In particular, the model of non-vesicular transport provides an attractive explanation for the strikingly low inner volume and protein concentration of the phagophore membrane.

A central question that arises from this model is whether Atg2–Atg18 bridges provide sufficient transfer capacity to explain the rapid and extensive growth of the phagophore membrane from a small vesicle to a double-membrane autophagosome. It seems clear from estimates that the transfer rate between vesicles mediated by Atg2–Atg18 *in vitro* is magnitudes to slow ^45^: an autophagosome can reach a diameter of 1 μm within a few minutes of biogenesis and thus contain around 25 million phospholipids requiring a total transfer rate of ≥50.000 phospholipid molecules * s ^−1^, but the extrapolated maximal transfer rate *in vitro* is only 0.017 phospholipid molecules * s ^−1^ per Atg2 molecule ^45^. Multiple Atg2 molecules contribute to the punctate structures seen at the ER–phagophore contact site by fluorescence light microscopy, but their number very likely cannot compensate for the low transfer rate *in vitro*. Thus, if Atg2-mediated transfer is the predominant way with which phospholipids are channeled from the ER into autophagic membrane assembly, mechanisms that dramatically accelerate phospholipid transfer via Atg2 *in vivo* must exist.

Very recent work has identified a pathway involving conserved acyl-coenzyme A synthetases (ACSs) and localized phospholipid synthesis in the ER that critically drives autophagic membrane assembly during phagophore expansion in yeast ^48^. ACSs are a conserved protein family of peripheral and transmembrane proteins that link coenzyme A to fatty acids to provide activated fatty acids for lipid synthesis, membrane editing, protein acylation, or vesicular fusion ^49^. In the context of autophagy, the conserved ACS Faa1 localizes to nucleated phagophores downstream of the assembled core autophagy proteins and locally activates fatty acids. These activated fatty acids are channeled into *de novo* phospholipid synthesis in the ER, which is essential for the efficient expansion of the phagophore and formation of functional autophagosomes ^48^. These data now show that newly synthesized phospholipids locally drive autophagic membrane formation. Thus, although a number of previously implicated organelles may contribute preformed membranes during phagophore expansion, they appear to be insufficient. These data indicate that localized *de novo* synthesis may constitute a driving force for the directed transfer of phospholipids across the Atg2–Atg18 complex from the ER into the membrane of the phagophore. Indeed, localized synthesis has been shown to accelerate the transfer of phospholipids across ER–mitochondria membrane contact sites ^50^. In line with these concepts, phospholipid-synthesizing enzymes appear to be enriched within the ER in proximity to forming autophagosomes and phosphatidylcholine synthesis promotes autophagy in mammalian cells ^51, 52^. In summary, although the mechanistic details have to be determined, the recent advances in our understanding of the mechanisms that drive the biogenesis of autophagosomes have shifted the focus to the principles of how cells modulate their lipid metabolism and transport across organelle contact sites in order to perform autophagy and maintain cellular health.
